# Safety and efficacy of early beta-blocker initiation in acute heart failure and cardiogenic shock: systematic review and meta-analysis

**DOI:** 10.1186/s43044-024-00558-3

**Published:** 2024-09-13

**Authors:** Cyndiana Widia Dewi Sinardja, Gusti Ngurah Prana Jagannatha, Bryan Gervais de Liyis, Anastasya Maria Kosasih

**Affiliations:** 1 Department of Cardiology and Vascular Medicine, Udayana University Hospital, Rumah Sakit Unud Street, Jimbaran, Badung, Bali Indonesia; 2 Faculty of Medicine, Udayana University/Prof. Dr. I.G.N.G Ngoerah General Hospital, Denpasar, Bali Indonesia

**Keywords:** Beta blocker, Acute heart failure, Cardiogenic shock, Mortality

## Abstract

**Background:**

The beta-blocker (BB) initiation in acute heart failure (AHF) patients is still controversial. Some show the benefit of BB employment in decreasing the mortality outcome. This study aims to assess the safety and efficacy of in-hospital and long-term outcomes of BB initiation in AHF hospitalized patients. We searched multiple databases examining the outcome of AHF patients who had administered BB as the therapy initiation. Primary outcomes were all-cause mortality, composite endpoint after BB initiation when hospitalized, and post-discharge all-cause mortality. The secondary outcomes were adverse events after in-hospital BB initiation, including hypotension and symptomatic bradycardia after BB initiation when hospitalization and rehospitalization.

**Results:**

Eight cohort studies with 16,639 patients suffering from AHF and cardiogenic shock, with 9923 participants allocated to the early BB group and 6,713 patients in the control group. The follow-up durations ranged from 2 to 24 months. Early BB administration significantly reduced in-hospital composite endpoints (RR: 0.42; 95% CI (0.30–0.58); *p* < 0.001), in-hospital all-cause mortality (RR: 0.43; 95% CI (0.31–0.61); *p* < 0.001), discharge mortality (RR: 0.51; 95% CI (0.41–0.63); *p* < 0.001), and rehospitalization (RR: 0.57; 95% CI (0.44–0.74); *p* < 0.001). There were no discernible differences in in-hospital BB-related adverse events between the two groups (*p* = 0.13). Subgroup analyses conducted on AHF patients presenting with cardiogenic shock revealed no significant differences in in-hospital composite endpoint and in-hospital mortality, and similar results were shown in the naive BB population.

**Conclusions:**

The BB initiation in AHF patients shows advantages in efficacy and safety outcome.

**Supplementary Information:**

The online version contains supplementary material available at 10.1186/s43044-024-00558-3.

## Background

The publication of some randomized controlled trials (RCT) these current days proved the effectiveness of beta blockers (BBs) in increasing the life expectancy of heart failure (HF) patients [1–4]. International guidelines recommended the employment of BB and renin-angiotensin system (RAAS) as the first-line treatment in chronic heart failure (CHF) patients [5]. The safety of using BB for HF patients shows a 30% decrease in mortality risk [6]. Regardless of the increasing prognosis of CHF, AHF is still a challenging situation in terms of treatment, which is fundamentally a symptomatic treatment. Recent data in Central Asia and Europe indicate that in-hospital mortality rates for AHF patients can reach 4–10%, with a one-year mortality rate ranging from 6.9 to 13% [7, 8].

The initiation of BB in AHF patients, whether the persistence of BB consumption for patients who used BB before or naive BB for first-time users, is still controversial and needs a further clinical assessment of BB. Currently, some studies show no benefits of short-term or long-term BB application [9]. However, another study shows the opposite result, which is the significant benefit of BB employment in long-term effect, even though the disappearance of protective effect after the treatment of covariate risk factor of classic HF [10].

Given the conflicting evidence, there is an urgent need for a large-scale empirical study to establish clear guidelines for BB initiation in AHF. This study aims to assess the safety and efficacy of BB initiation during hospitalization and its long-term outcomes in patients with AHF.

## Methods

This systematic review adhered to the rigorous methodology outlined in the Preferred Reporting Items for Systematic Reviews and Meta-analyses (PRISMA) guidelines, ensuring transparency and quality in reporting [11].

### Search strategy and selection criteria

A comprehensive search strategy was employed to identify relevant studies for inclusion in this systematic review. The following electronic databases were searched without language restrictions: MEDLINE (Medical Literature Analysis and Retrieval System Online) through PubMED, EMBASE (Excerpta Medical Database), and Cochrane Library. The search covered the period from the inception of these databases until November 17, 2023. The search strings used were: ((Acute Heart Failure) or (Acute Decompensated Heart Failure) or (Cardiogenic Shock)) and ((Major Adverse Cardiac Event) and (Mortality) or (Side effect)). Additionally, citation tracking was conducted to identify any additional relevant publications that may have been missed through the database search.

All identified studies were screened by title and abstract. Three researchers independently identified studies that met the inclusion criteria (G.N.P.J., B.G.L., and A.M.K.). Any discrepancies or disagreements were resolved through consensus discussions among the researchers. The inclusion criteria for this meta-analysis were studies examining the outcome of BB initiation during hospitalization in the acute phase and prior to discharge in AHF patients, including de novo AHF, acute decompensated heart failure (ADHF), and cardiogenic shock. These criteria are based on international guidelines [5]. The exclusion criteria were criteria diagnoses that were not suitable with guidelines [5] and unclear of BB initiation onset. Final eligibility was decided after the evaluation of full-text publication. All disagreements are settled through discussion or involving a fourth referee (C.W.S). Data extraction and quality assessment.

Data extraction and quality assessment data extraction were carried out independently by three researchers (G.N.P.J., B.G.L., and A.M.K.), and all disagreements were settled through discussion or with the involvement of a fourth referee (C.W.S). Standard forms were used to extract the following information from each study: (i) study design and methodology; (ii) type of AHF; (iii) specific condition of AHF (Naïve BB, cardiogenic shock, or not specified); (iv) type and dose of BB; (v) baseline characteristics; and (vi) outcome as stated in the protocol of the current meta-analysis. Upon identifying any issues with the main results, such as missing data or unclear information, the authors of the original publication were promptly notified via email. This communication aimed to address and clarify any discrepancies or uncertainties encountered during the review process.

Three researchers (G.N.P.J., B.G.L., and A.M.K.) independently evaluated the included papers' systematic quality using the recommended Newcastle–Ottawa scale (NOS) for observational studies [12]. Investigations were classified as having low (< 5 points), moderate (5–7 points), and high quality (> 7 points), and any differences were settled through discussion or by involving a fourth referee (C.W.S).

### Outcome measurement

The primary outcomes of this analysis were all-cause mortality, the composite endpoint after BB initiation during hospitalization (including total all-cause mortality, cardiovascular death, myocardial infarction, stroke, ventricular arrhythmia, and ventilator support), and post-discharge all-cause mortality. Subgroup analyses were conducted on all primary outcome parameters for patients who experienced cardiogenic shock and those who were first-time BB users. The secondary outcomes included in-hospital BB-related adverse events, such as hypotension and symptomatic bradycardia following BB initiation during hospitalization, as well as rates of rehospitalization.

### Data synthesis and analysis quality assessment

Data for a specific variable were included in the synthesis if it was reported in at least two of the included studies. Heterogeneity between the study populations was assessed using the *I*^2^ statistic [13]. Heterogeneity levels were classified as low, medium, and high when the *I*^2^ values were less than or equal to 25%, 50%, and 75%, respectively. Data across groups were summarized using the Mantel–Haenszel (MH) risk ratio (RR) fixed-effect model if *I*^2^ < 25%. For *I*^2^ values greater than 25%, the random-effect model was employed [14]. All analyses were conducted with 95% confidence intervals (95% CI). Funnel plots were used to evaluate publication bias as previously described [14, 15]. Analysis was carried out using Review Manager 5.4.

## Results

### Study selection and risk of bias

**Fig. 1:**
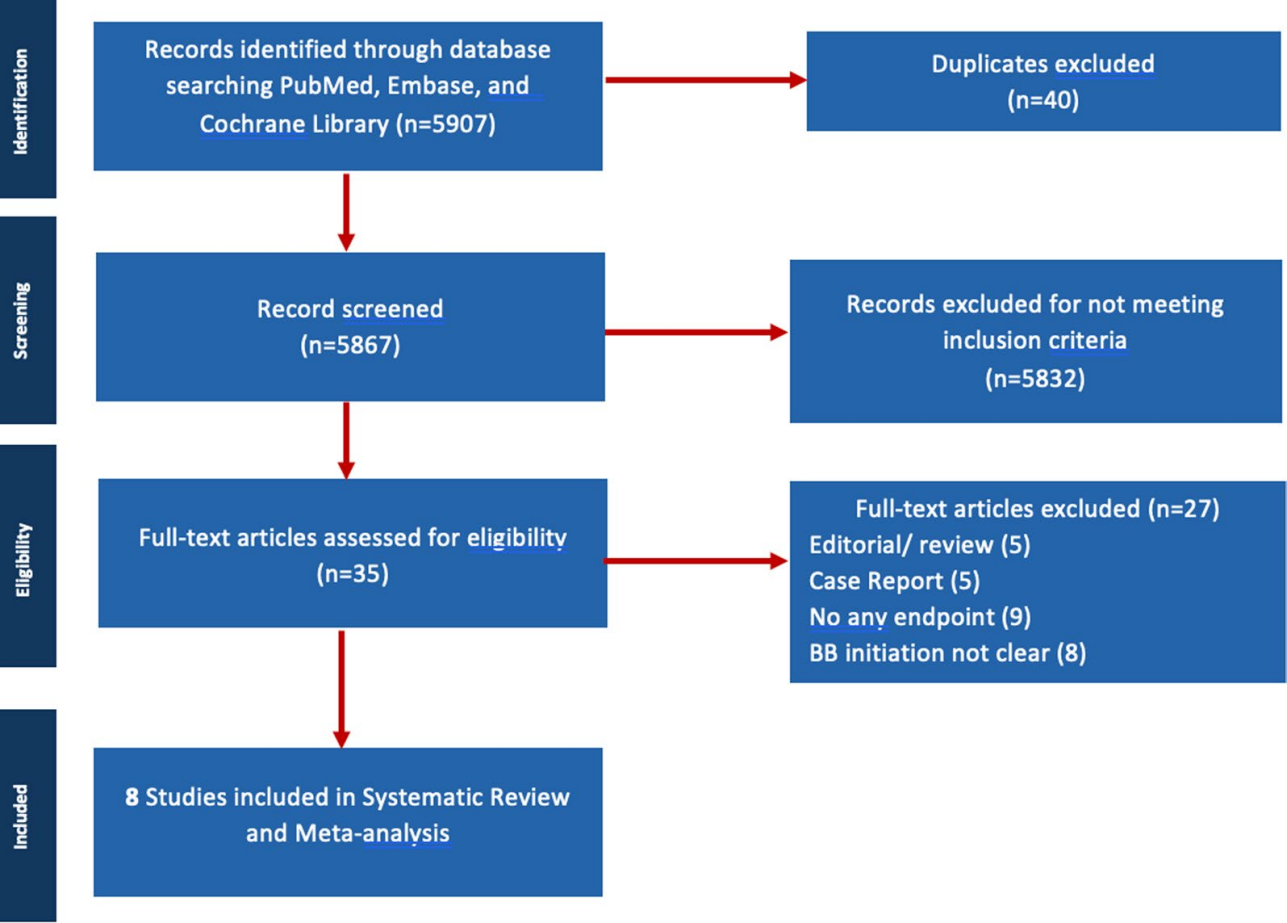
Preferred reporting items for systematic reviews and meta-analyses (PRISMA) flow diagram

After screening 5,907 studies from electronic databases, 40 were excluded due to duplication, irrelevant populations, outcomes, and methods. Ultimately, 35 studies met the eligibility criteria and were included in the meta-analysis. The study selection and data extraction process followed the PRISMA guidelines, as shown in Fig. 1. Risk of bias was assessed using the NOS tool for 8 cohort studies (Supplementary Table 1). To evaluate the impact of publication bias, we performed a funnel plot analysis, which is visually presented in Supplementary Figs. 1–9. Ideally, a balanced distribution of data points around the mean effect size suggests no publication bias, while an uneven distribution may indicate its presence.

### Characteristics of included studies

The characteristics of the included studies are summarized in Table 1. These studies involved 16,639 patients with acute heart failure AHF and cardiogenic shock. Of these, 9,923 patients were assigned to the early BB group, and 6,713 patients were in the control group. The cohort had 60.38% with coronary artery disease (CAD), 41.43% with hypertension (HT), and 34.37% with diabetes mellitus (DM). Follow-up durations ranged from 2 to 24 months, providing insight into long-term outcomes.

**Table 1:** Baseline characteristics

Study, year	Total population	BB group	Control group	Baseline BB use	CAD (%)	HT (%)	DM (%)	Follow-up period	CS	Timing of early BB initiation
Bohm et al., 2011	1104	805	299	589	66.9	11.7	33.3	6 months	No	BB continued during hospitalization, initiated at discharge for new patients
Butler et al., 2006	263	209	54	268	44.7	46.4	33.8	6 months	No	BB initiated at discharge
Cho et al., 2018	672	282	390	NA	49.2	47.5	39.4	24 months	Yes	BB initiated upon admission
Fonarow et al., 2008	2373	1982	382	1429	60.75	66.2	26	2 months	No	BB initiated during hospitalization, specific timing varies
Orso et al., 2009	1572	620	952	503	43.8	NA	41.4	NA	NA	BB continued during hospitalization, initiated at discharge for new patients
Ryu et al., 2022	224	143	84	NA	57.3	67.4	47.1	NA	Yes	BB initiated during hospitalization alongside vasopressors and inotropes
Santo et al., 2021	192	93	99	NA	NA	NA	NA	NA	Yes	BB initiated upon admission
Wang et al., 2022	10,239	5789	4450	NA	100	48.5	19.6	NA	No	BB initiated during hospitalization, specific timing varies

*BB* Beta blocker, *CAD* Coronary artery disease, *CS* Cardiogenic shock, *DM* Diabetes mellitus, *HT* Hypertension

### Primary and secondary endpoints

**Fig. 2:**
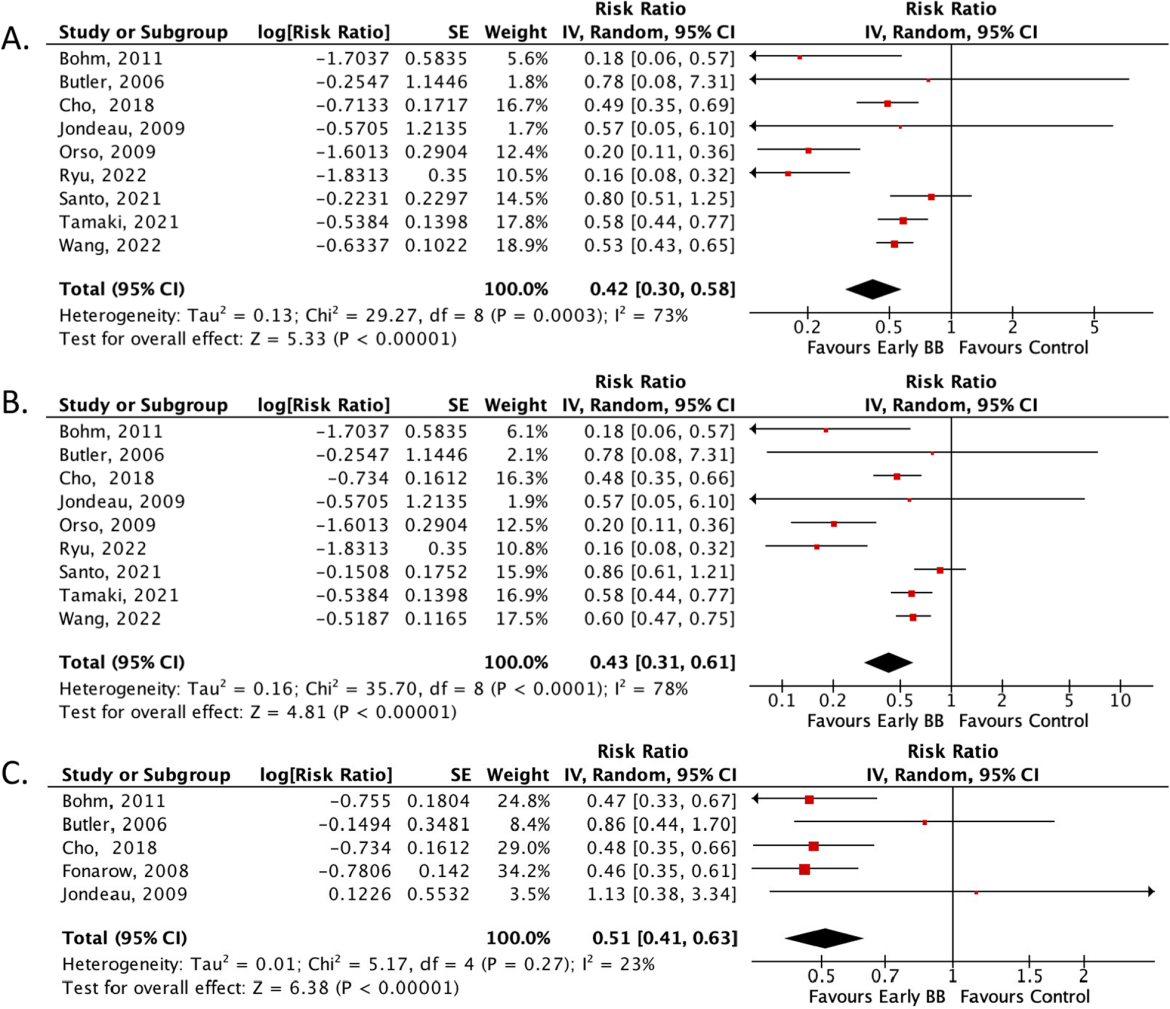
Forrest plot of primary outcomes. **A** Risk ratio of in-hospital composite endpoint; **B** risk ratio of in-hospital all-cause mortality; **C** risk ratio of post-discharge mortality. *CI* Confidence interval, *MH* Mantel–Haenszel, *SE* Size effect

**Fig. 3:**
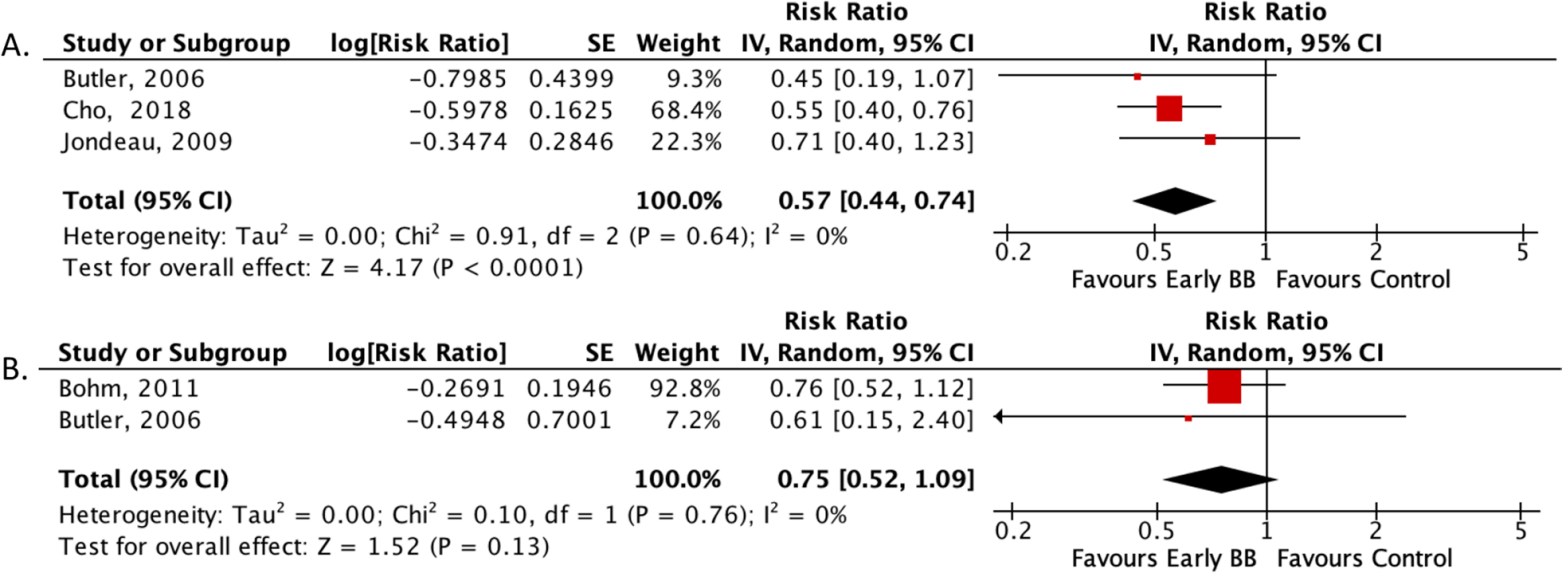
Forrest plot of secondary outcomes. **A** Risk ratio of rehospitalization; **B** risk ratio of in-hospital BB-related adverse events. *CI* Confidence interval, *MH* Mantel–Haenszel, *SE* Size effect

Our analysis showed that early BB administration significantly reduced in-hospital composite endpoints (RR: 0.42; 95% CI 0.30–0.58; *p* < 0.001; *I*^2^** = **73%; Fig. 2A), in-hospital all-cause mortality (RR: 0.43; 95% CI 0.31–0.61; *p* < 0.001; *I*^2^ = 78%; Fig. 2B), discharge mortality (RR: 0.51; 95% CI 0.41–0.63; *p* < 0.001; *I*^2^ = 23%; Fig. 2C), and rehospitalization (RR: 0.57; 95% CI 0.44–0.74; *p* < 0.001; *I*^2^ = 0%; Fig. 3A). There were no significant differences in BB-related adverse events, including bradyarrhythmias and hypotension, were not significantly different between the groups during hospitalization (RR: 0.75; 95% CI 0.52–1.09; *p* = 0.13; *I*^2^ = 0%; Fig. 3B).

### Subgroup analyses

**Fig. 4:**
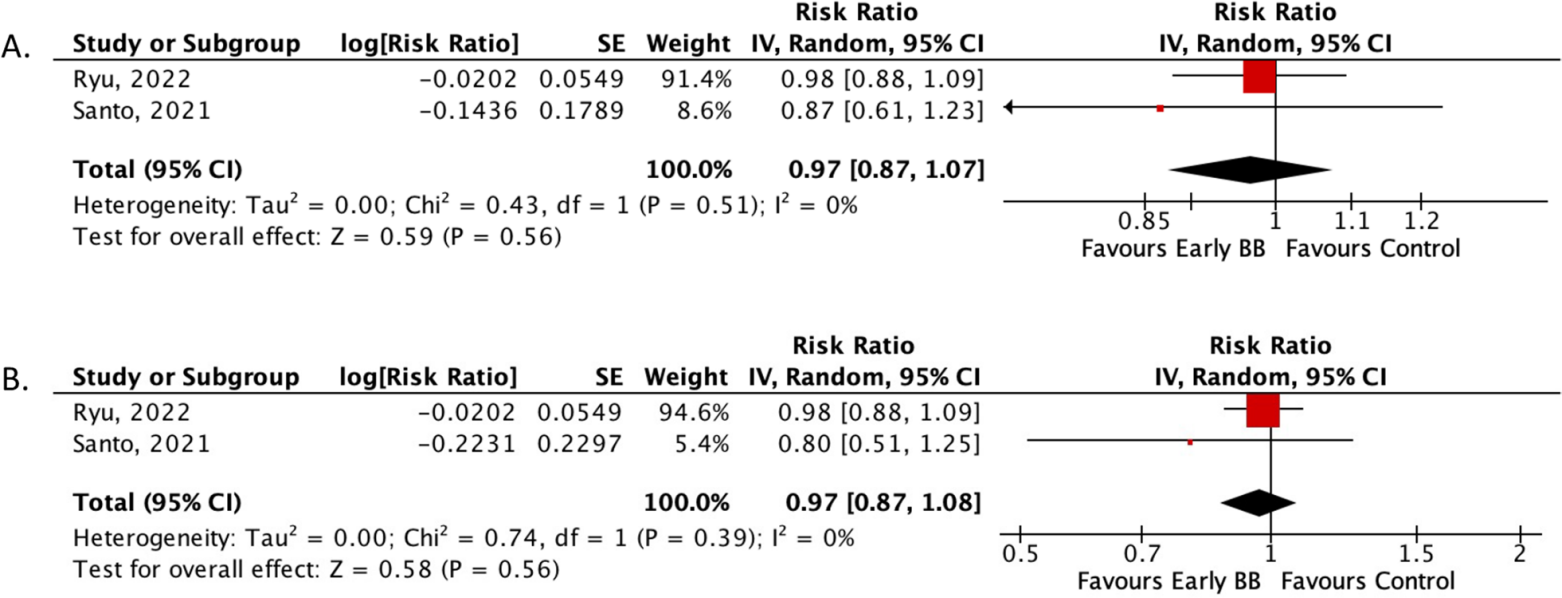
Forrest plot subgroup analysis based on cardiogenic shock occurrence. **A** Risk ratio of in-hospital composite endpoint; **B** risk ratio of in-hospital all-cause mortality. *CI* Confidence interval, *MH* Mantel–Haenszel, *SE* Size effect

**Fig. 5:**
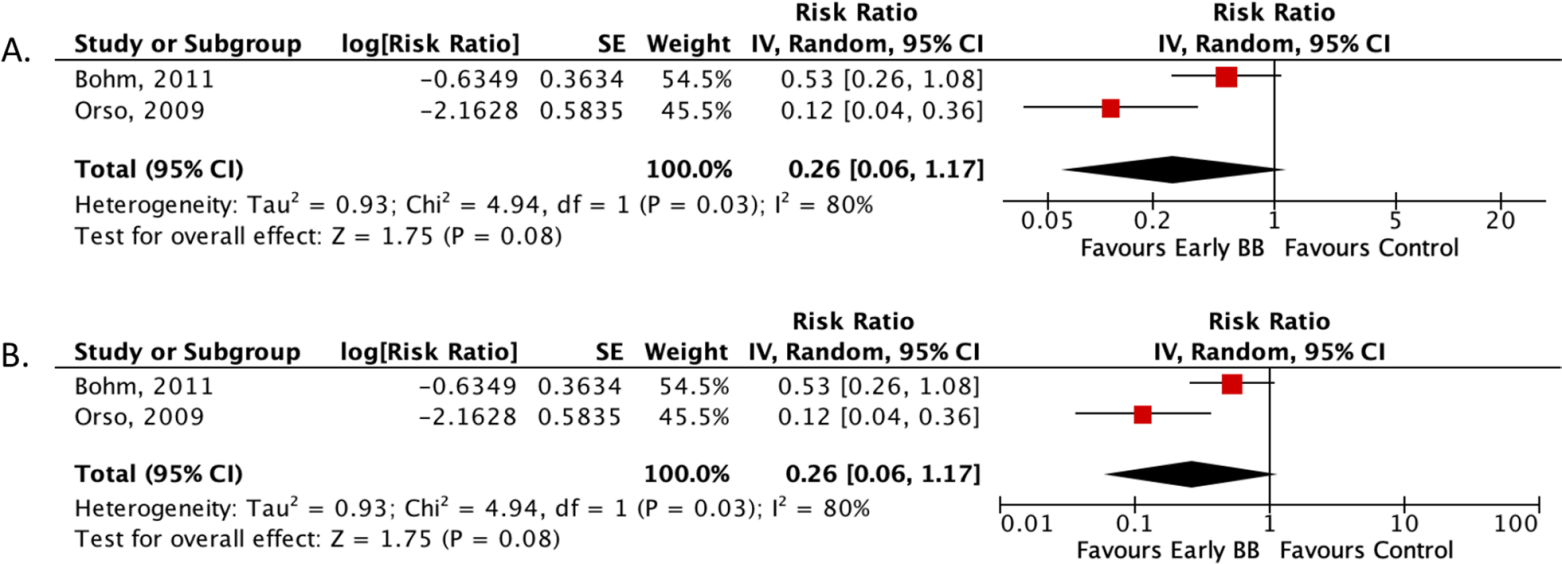
Forrest plot subgroup analysis based on naïve BB in AHF patients. **A** Risk ratio of in-hospital composite endpoint; **B** risk ratio of in-hospital all-cause mortality. *CI* Confidence interval, *MH* Mantel–Haenszel, *SE* Size effect

Subgroup analyses provided further insight into specific patient populations. In AHF patients with cardiogenic shock, no significant differences were observed in the in-hospital composite endpoint (RR: 0.97; 95% CI 0.87–1.08; *p* = 0.56) or in-hospital mortality (RR: 0.97; 95% CI 0.87–1.07; *p* = 0.56) between the early BB administration group and the control group (Fig. 4A, B). Similarly, in patients naive to BB treatment, both the in-hospital composite endpoint (RR: 0.26; 95% CI 0.06–1.17; *p* = 0.08) and in-hospital mortality (RR: 0.26; 95% CI 0.06–1.17; *p* = 0.08) did not show significant differences (Fig. 5A, B). These findings suggest that the benefits of early BB administration may not extend uniformly across all patient subgroups, particularly those with cardiogenic shock and BB-naive patients.

## Discussions

The significant reduction in in-hospital composite endpoints, all-cause mortality, discharge mortality, and rehospitalization underscores the potential therapeutic efficacy of initiating BBs early in the course of these conditions. The heterogeneity observed in our meta-analysis, as indicated by the *I*^2^ statistics, highlights the variability in patient populations, treatment regimens, and study designs across the included studies. For example, the moderate to high *I*^2^ values for in-hospital composite endpoints and all-cause mortality suggest that the effects of early BB administration might be influenced by factors such as differences in baseline patient characteristics, the specific types and doses of BBs used, and variations in clinical practice. While the heterogeneity does not negate the overall positive findings, it does suggest that the effectiveness of BBs may vary depending on specific clinical contexts. Conversely, the lower *I*^2^ values observed for discharge mortality and rehospitalization indicate more consistent effects across studies for these outcomes. This consistency strengthens the evidence that early BB initiation has a robust and reliable impact on reducing these specific endpoints, regardless of the study population or treatment variations.

The early initiation of BBs in the setting of AHF may influence several pathophysiological processes, thereby mitigating in-hospital composite endpoints, such as need for ventilation support, cardiac arrest, and arrhythmia malignant. BBs can positively impact ventricular function, especially in the setting of heart failure. By antagonizing beta receptors, these medications may enhance left ventricular ejection fraction, reduce chamber dilation, and improve overall cardiac performance [16, 17]. This improvement in cardiac function contributes to a decreased need for ventilation support and may prevent the progression to severe complications [18]. The reduction in in-hospital composite endpoints suggests that early BB administration contributes to a more favorable overall clinical course during the hospitalization period. Early BB administration may contribute to the prevention of cardiac ischemia, a common complication in AHF. By reducing myocardial oxygen demand and improving coronary blood flow, BB helps maintain optimal myocardial function, lowering the risk of events such as cardiac arrest [19, 20]. BBs antagonize the effects of sympathetic nervous system activation by blocking beta-adrenergic receptors. In the context of AHF, where sympathetic overactivity is often prevalent, early administration of BBs can attenuate the excessive release of catecholamines [21]. This modulation contributes to a rebalance in the neurohormonal environment, mitigating oxidative stress—a hallmark of AHF. Additionally, some BBs exhibit antioxidant and anti-inflammatory properties, potentially mitigating cellular damage and systemic inflammation associated with heart failure [22].

BBs further exhibit a dual role by reducing the production of reactive oxygen species (ROS) by modulating mitochondrial bioenergetics, pivotal in preventing oxidative damage to cardiac tissues [23]. This reduction in oxidative stress is complemented by the anti-inflammatory effects of BBs, as they downregulate inflammatory pathways, providing a comprehensive shield against cellular damage. This has the potential to decrease the strain on the heart, lower myocardial oxygen demand, and prevent the development of malignant arrhythmias [24]. The diminished in-hospital all-cause mortality and discharge mortality further support the notion that early BB intervention may confer a survival benefit and improve outcomes at the point of hospital discharge. Similarly, a recent study conducted by Tamaki et al. revealed a significant association between BB use at admission and a reduced risk of in-hospital mortality (odds ratio, 0.41; 95% CI 0.27–0.60, *p* < 0.001) [25]. These additional benefits contribute to a favorable overall impact on mortality outcomes. The lower rates of rehospitalization in the early BB group imply a sustained positive impact beyond the initial hospitalization, reflecting a potential long-term benefit associated with early BB initiation.

Notably, the absence of discernible differences in in-hospital BB-related adverse events between the early BB group and the control group suggests that the observed benefits were achieved without a significant increase in immediate adverse effects associated with BB therapy. This suggests that the observed benefits associated with early BB initiation, such as a significant reduction in in-hospital composite endpoints, all-cause mortality, discharge mortality, and rehospitalization, were achieved without exposing patients to a heightened risk of immediate adverse effects commonly associated with BB therapy. BBs, known for their efficacy in heart failure management, may pose concerns related to adverse events, including bradycardia, hypotension, and bronchospasm [13]. The lack of a significant difference in adverse events suggests that the benefits of early BB administration in AHF are realized without an undue burden of immediate safety concerns. Moreover, Liang et al. demonstrated that BBs use had no significant long-term effect on the risk of hospitalization for HF, recurrent MI, stroke, or repeat revascularization in post-MI patients [26].

The subgroup analyses for patients with cardiogenic shock and BB-naive patients revealed no significant differences in in-hospital composite endpoints or mortality when comparing early BB administration with standard care. These findings suggest that the benefits observed in the broader AHF population may not extend uniformly to all patient subgroups. The lack of significant benefit in patients with cardiogenic shock may be attributed to the unique pathophysiological challenges in this group, such as severe hemodynamic instability that requires immediate and aggressive management [27]. The use of BBs in this context might be limited due to the need for inotropes and vasopressors, which are essential for maintaining perfusion but may counteract the effects of BBs [27, 28]. As a result, while BBs may be beneficial in more stable phases of treatment, their initiation during the acute phase of cardiogenic shock might not provide the same advantages as observed in the general AHF population. This underscores the importance of individualized treatment strategies, where the timing of BB initiation is carefully considered in relation to the patient’s hemodynamic status. Similarly, the absence of significant differences in outcomes for BB-naive patients suggests that the initiation of BBs during acute decompensation may not yield immediate benefits in this subgroup. The therapeutic effects of BBs typically require time to manifest, and patients without prior BB exposure might not experience the immediate stabilization benefits seen in those with long-term BB therapy [29, 30]. This finding highlights the need for further research to optimize the timing and dosing of BBs in beta-blocker-naive patients, as well as to explore potential strategies for gradually introducing BB therapy in this population.

The findings of this study underscore the potential of early BB administration as a transformative approach in the management of AHF. By significantly improving in-hospital outcomes, reducing mortality, and lowering rehospitalization rates without increasing adverse events, early BB therapy could shift current treatment paradigms. These results suggest that incorporating early BB initiation into clinical guidelines could enhance patient outcomes, particularly by stabilizing high-risk patients early in their hospital course. Furthermore, the demonstrated safety in subgroups like those with cardiogenic shock and BB-naive patients highlights the versatility of BBs, suggesting that they could be safely expanded to broader patient populations. This study supports a move toward more personalized and proactive AHF management strategies, potentially leading to improved long-term patient survival and reduced healthcare costs through fewer rehospitalizations.

While our meta-analysis provides valuable insights, several limitations should be noted. First, the analysis is based solely on cohort studies, which are prone to selection bias and may not establish causality as robustly as RCTs. The inherent variability in study methodologies, patient populations, and beta-blocker types and doses contributes to heterogeneity in the results, as indicated by the moderate to high *I*^2^ values. This variability limits the generalizability of our findings across all AHF populations. Additionally, the definition of "early" BB initiation varied among the included studies. Some studies defined early initiation as the continuation of BB therapy during hospitalization, others as starting BB therapy at discharge, and some as immediate initiation upon admission. This variation in timing may influence the outcomes observed in different clinical contexts. For instance, immediate BB initiation might provide early stabilization benefits but could pose risks in patients with severe hemodynamic instability, such as those in cardiogenic shock. Conversely, initiation during hospitalization after initial stabilization might balance the risks and benefits, while initiation at discharge could help prevent post-discharge complications but might miss the opportunity to stabilize the patient during the acute phase of hospitalization. These differences in timing could affect the interpretation of our results and highlight the need for individualized treatment strategies. Moreover, the follow-up periods in the included studies ranged from 2 to 24 months, which may be insufficient to fully assess long-term effects and safety. Finally, the limited data available for specific subgroups, such as patients with cardiogenic shock, further restrict the applicability of our conclusions. Future research, particularly well-designed RCTs with extended follow-up, is needed to confirm the benefits and long-term outcomes of early beta-blocker initiation in diverse clinical settings.

The included studies in our meta-analysis span multiple regions, including Asia, Europe, and North America, each with distinct healthcare systems and practices. One notable difference is the length of hospital stays, which tend to be longer in some Asian countries, such as Japan, compared to Western countries [31]. This difference in hospitalization practices could impact the timing of interventions, including BB initiation, as well as the monitoring and management of adverse events. Longer hospital stays might allow for more gradual initiation and titration of BBs, potentially leading to different outcomes compared to settings where shorter hospital stays are the norm. These geographical and healthcare system differences may affect the generalizability of our findings. For instance, the benefits observed in settings with longer hospital stays might not be directly applicable to healthcare systems where early discharge is prioritized. Additionally, variations in healthcare access, resource availability, and clinical guidelines across regions could influence treatment decisions and outcomes. Therefore, while our meta-analysis suggests overall benefits of early BB initiation in acute heart failure, the applicability of these findings across different healthcare settings should be considered with caution.

## Conclusions

The BB initiation in AHF patients shows advantages in efficacy and safety by reducing the in-hospital composite endpoints, in-hospital all-cause mortality, discharge mortality, and rehospitalization. Therefore, BB initiation should be recommended as early as possible in AHF patients.

## Supplementary Information

Additional file 1.

## Data Availability

All data on this meta-analysis are included in the published article.

## Abbreviations

BB: Beta blocker
AHF: Acute heart failure
CS: Cardiogenic shock
HF: Heart failure
RCT: Randomized controlled trial
CHF: Congestive heart failure
PRISMA: Preferred reporting items for systematic reviews and meta-analyses
ADHF: Acute decompensated heart failure
NOS: Newcastle–Ottawa scale
