# Supplementary material for: Safety and efficacy of early beta-blocker initiation in acute heart failure and cardiogenic shock: systematic review and meta-analysis
Source: Egypt Heart J. 2024 Sep 13;76:126. doi: 10.1186/s43044-024-00558-3 (PMC11399533; doi:10.1186/s43044-024-00558-3)

**SUPPLEMENTARY MATERIAL**

**Supplementary Table 1:** Risk of bias assessment of observational studies included in the meta-analysis according to the Newcastle-Ottawa Scale

| **Study** | **Selection** | | | | **Comparability** | | **Outcome** | | | **Total** |
| --- | --- | --- | --- | --- | --- | --- | --- | --- | --- | --- |
|  | **1** | **2** | **3** | **4** | **1** | **2** | **1** | **2** | **3** |  |
| Bohm et al, 2011 | $*$ | $*$ | $*$ | $*$ | $*$ | **-** | $*$ | $*$ | $*$ | 8 |
| Butler et al, 2006 | $*$ | $*$ | $*$ | $*$ | **-** | **-** | $*$ | $*$ | $*$ | 7 |
| Cho et al, 2018 | $*$ | $*$ | $*$ | $*$ | **-** | **-** | $*$ | $*$ | $*$ | 6 |
| Fonarow et al, 2008 | $*$ | $*$ | $*$ | $*$ | $*$ | **-** | **-** | $*$ | $*$ | 7 |
| Orso et al, 2009 | $*$ | $*$ | $*$ | $*$ | **-** | **-** | **-** | $*$ | $*$ | 6 |
| Ryu et al, 2022 | $*$ | $*$ | $*$ | $*$ | **-** | **-** | $*$ | $*$ | $*$ | 7 |
| Santo et al, 2021 | $*$ | $*$ | $*$ | $*$ | $*$ | $*$ | $*$ | $*$ | $*$ | 9 |
| Wang et al, 2022 | $*$ | $*$ | $*$ | $*$ | $*$ | **-** | $*$ | $*$ | $*$ | 8 |

**Supplementary Figure 1.** Funnel plot of in-hospital all cause morality


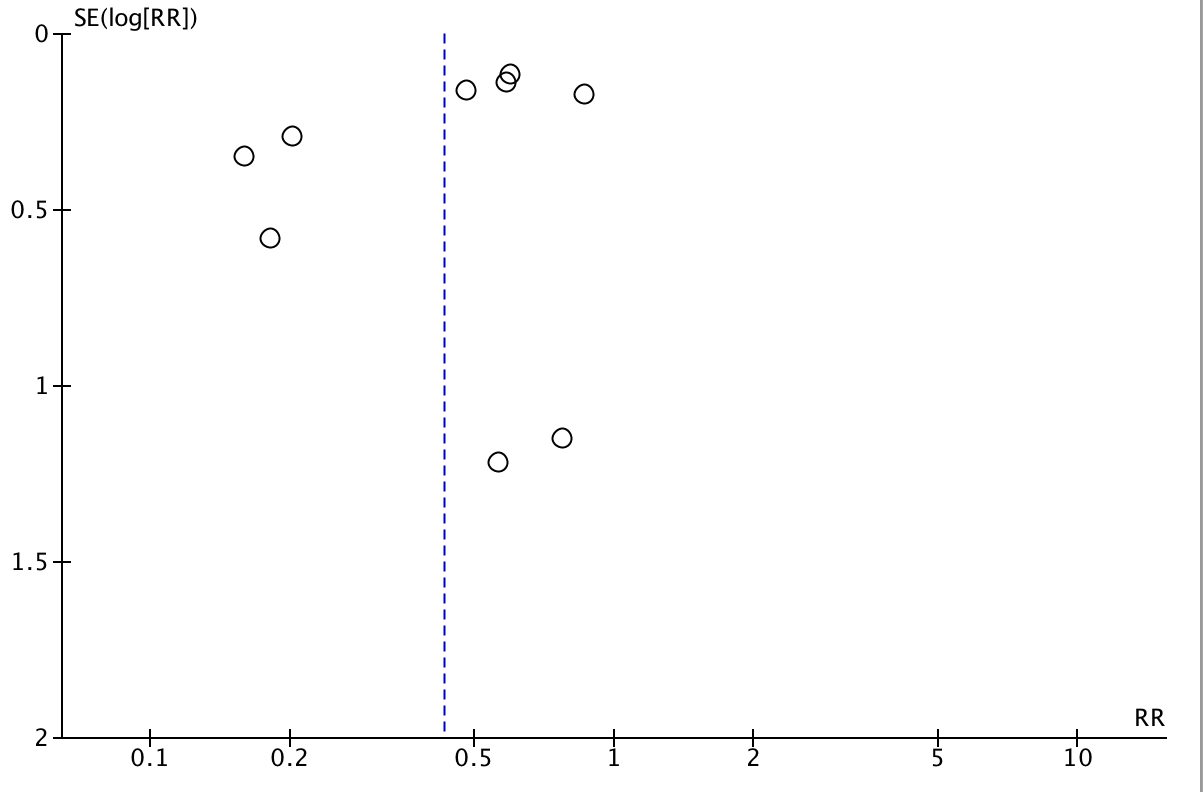


**Supplementary Figure 2.** Funnel plot of in-hospital composite endpoint


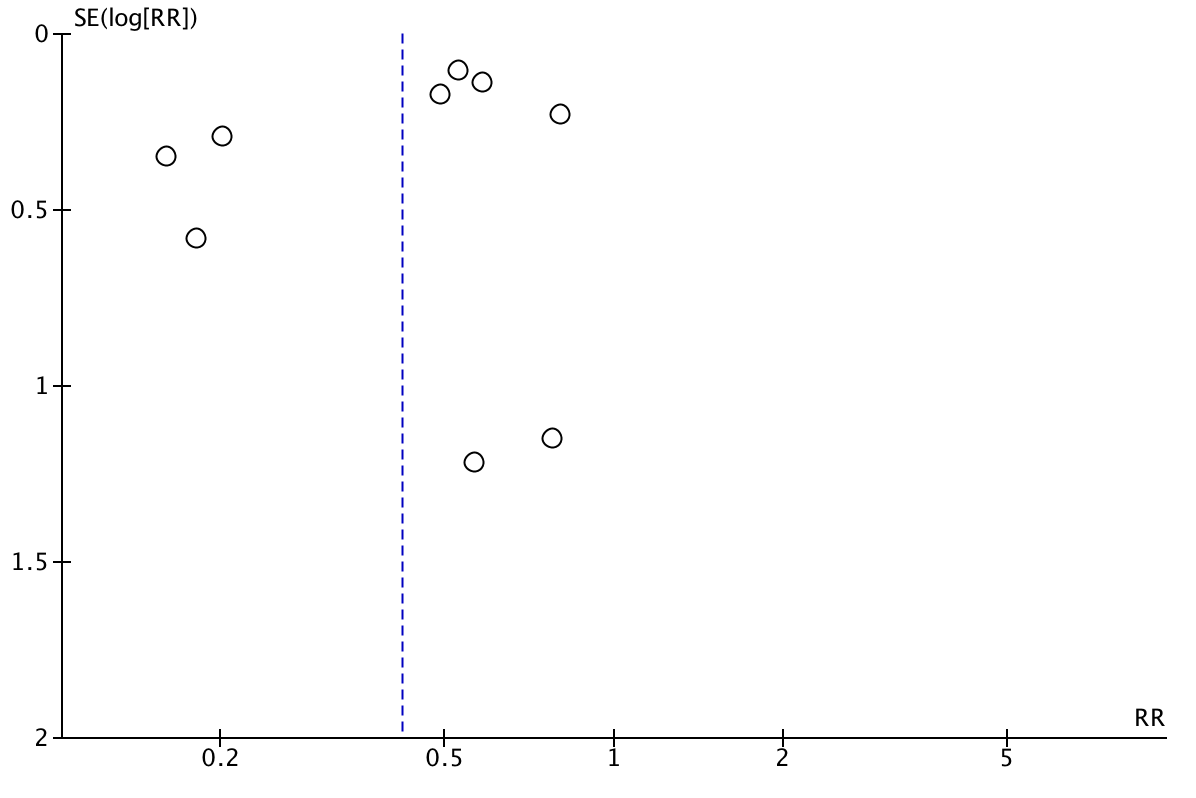


**Supplementary Figure 3.** Funnel plot of in-hospital Beta blocker related adverse events


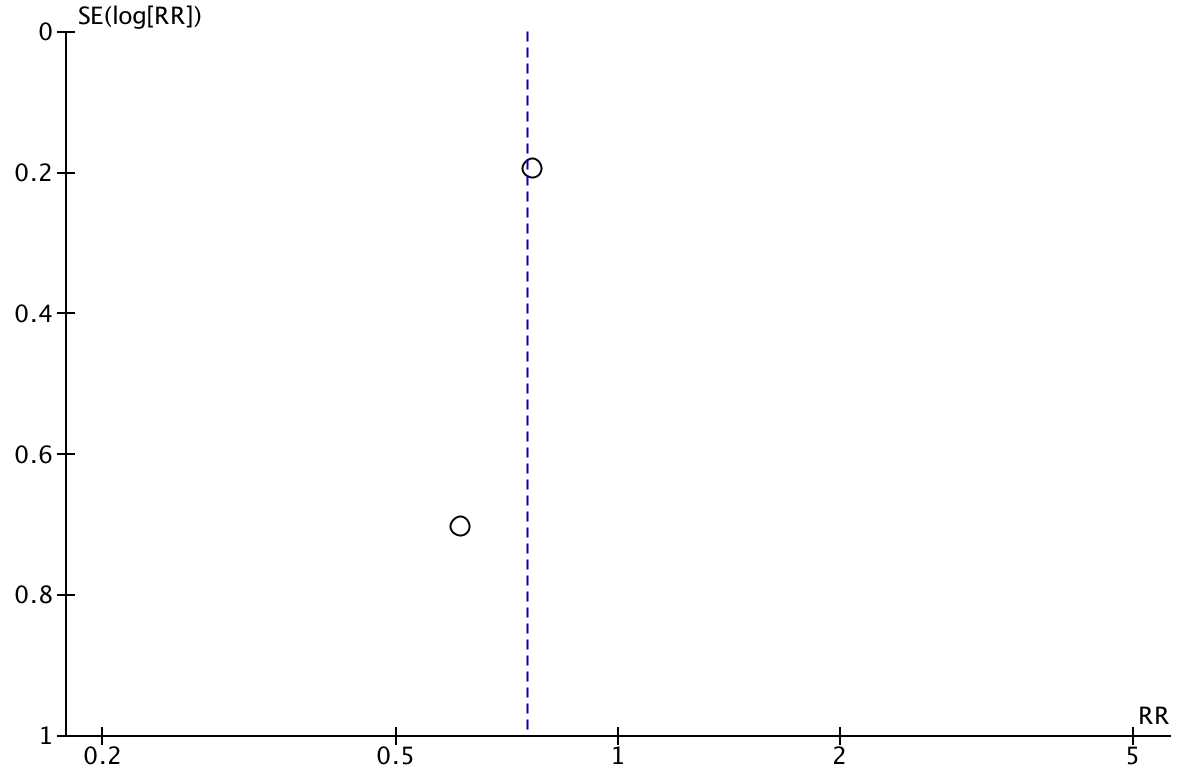


**Supplementary Figure 4.** Funnel plot of post-discharge mortality


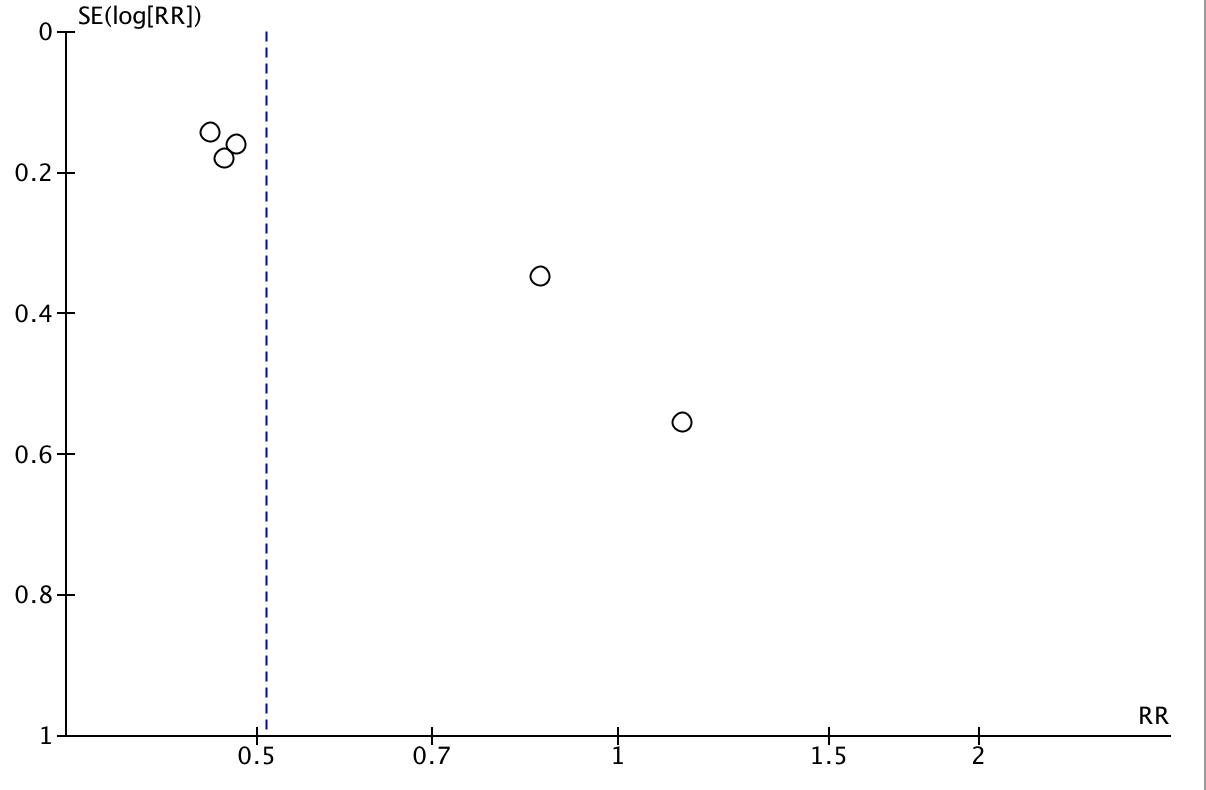


**Supplementary Figure 5.** Funnel plot of re-hospitalization


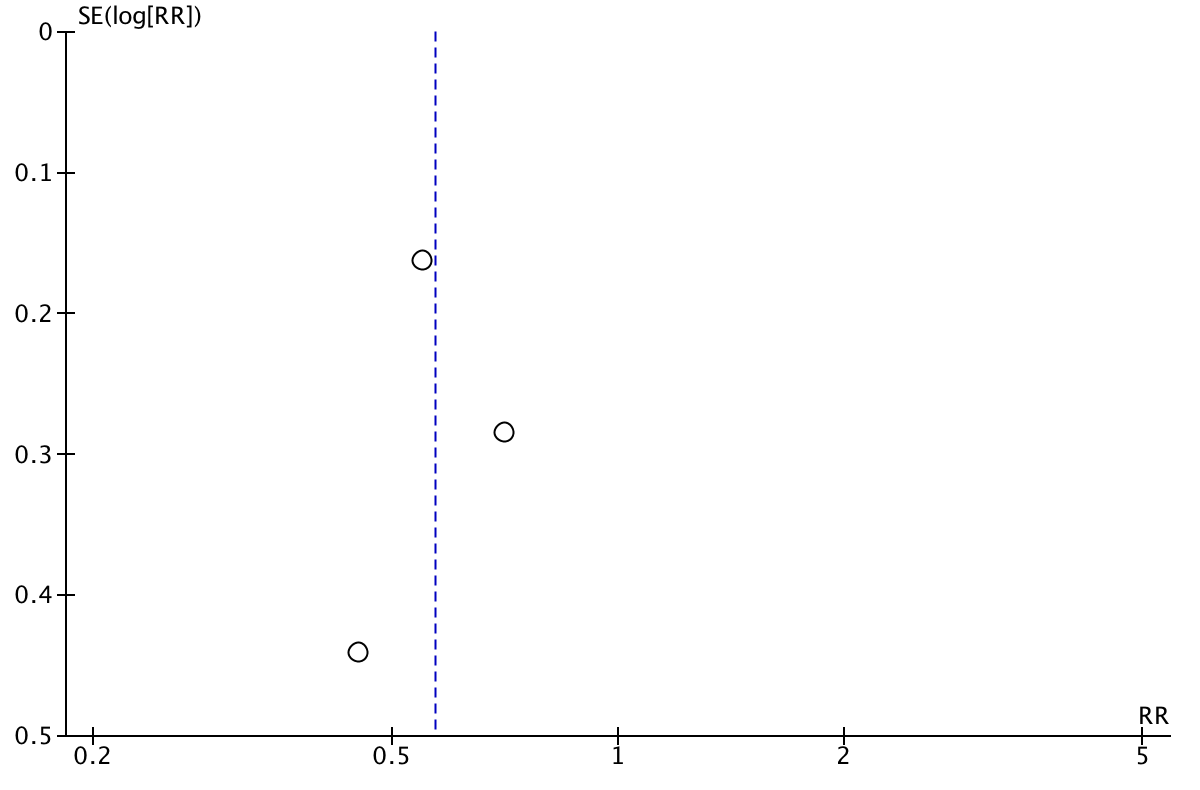


**Supplementary Figure 6.** Funnel plot of in-hospital composite endpoint based on cardiogenic shock subgroup


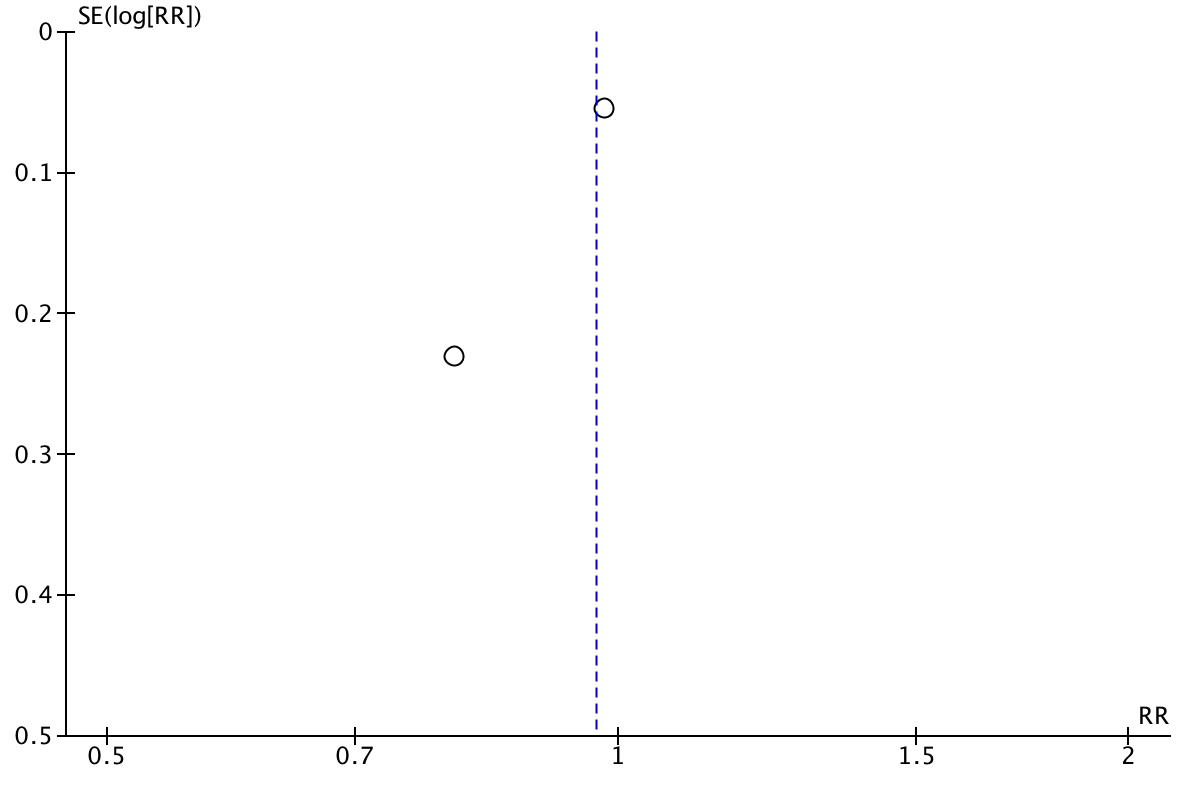


**Supplementary Figure 7.** Funnel plot of in-hospital all-cause mortality based on cardiogenic shock subgroup


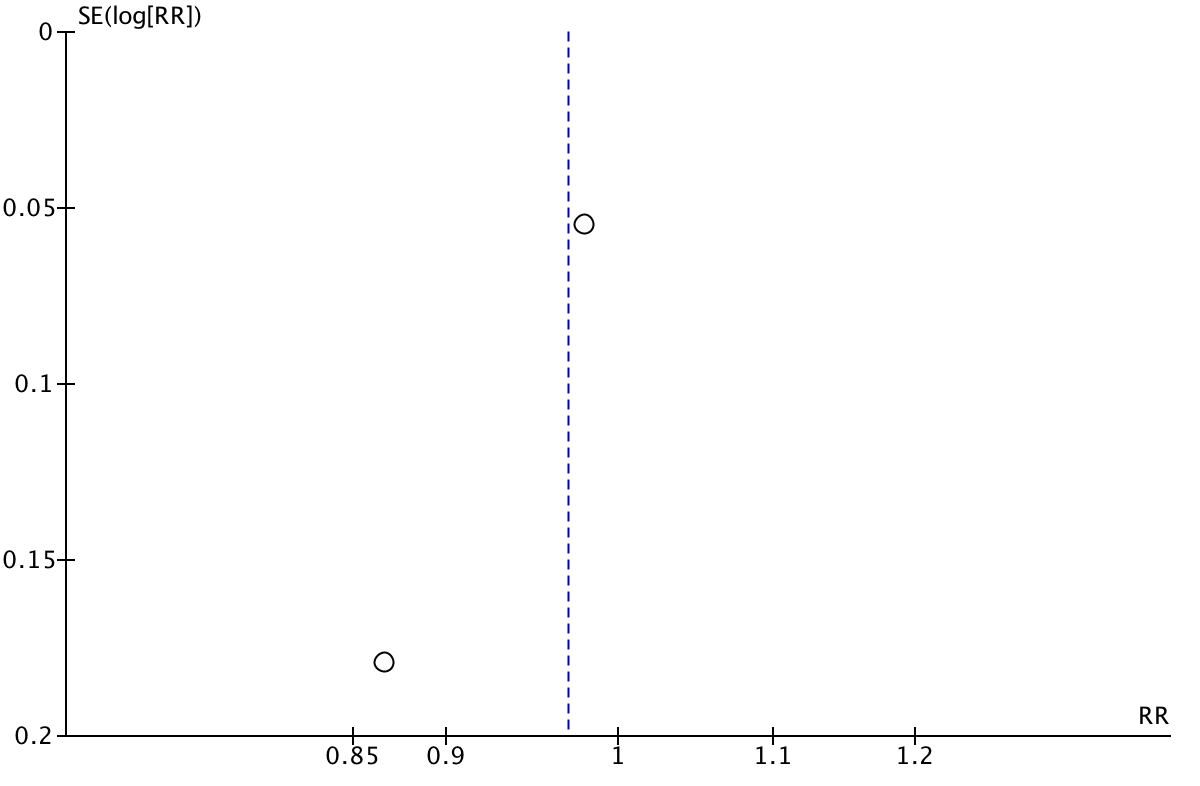


**Supplementary Figure 8.** Funnel plot of in-hospital composite endpoint based on naïve beta blocker subgroup


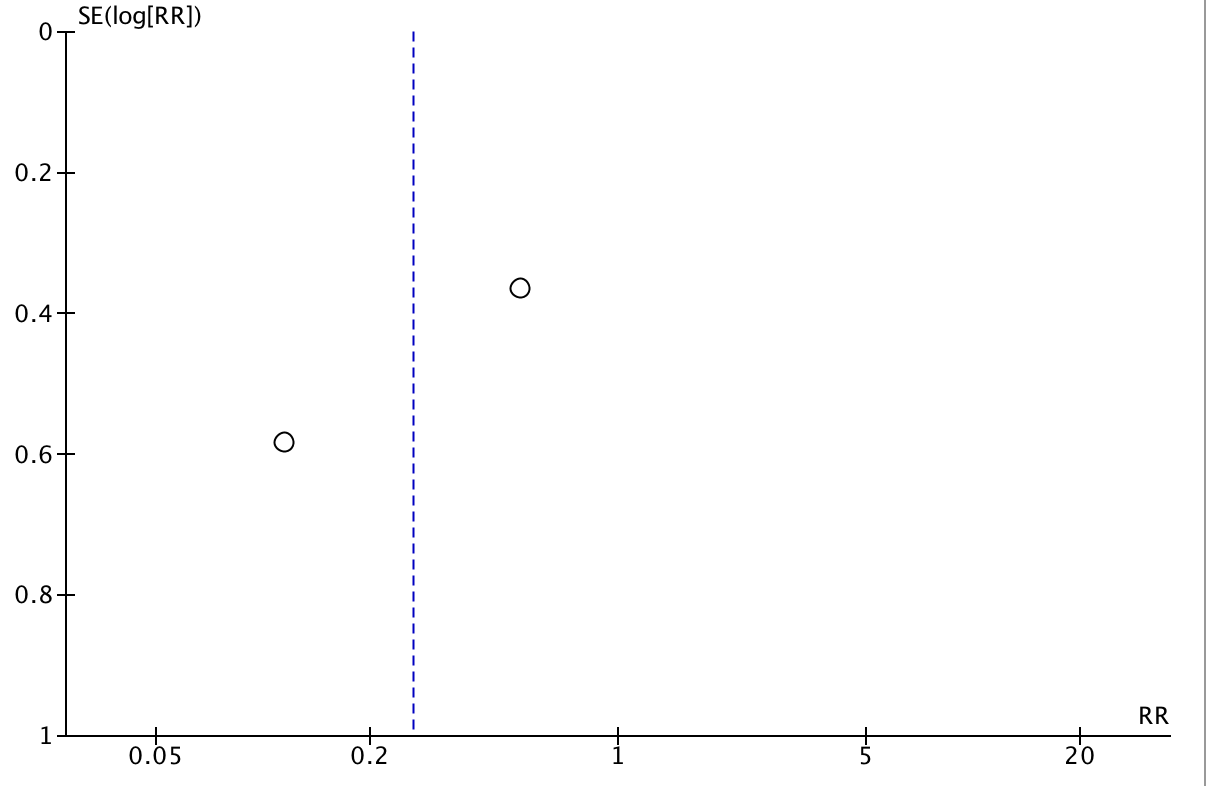


**Supplementary Figure 9.** Funnel plot of in-hospital all-cause mortality based on naïve beta blocker subgroup


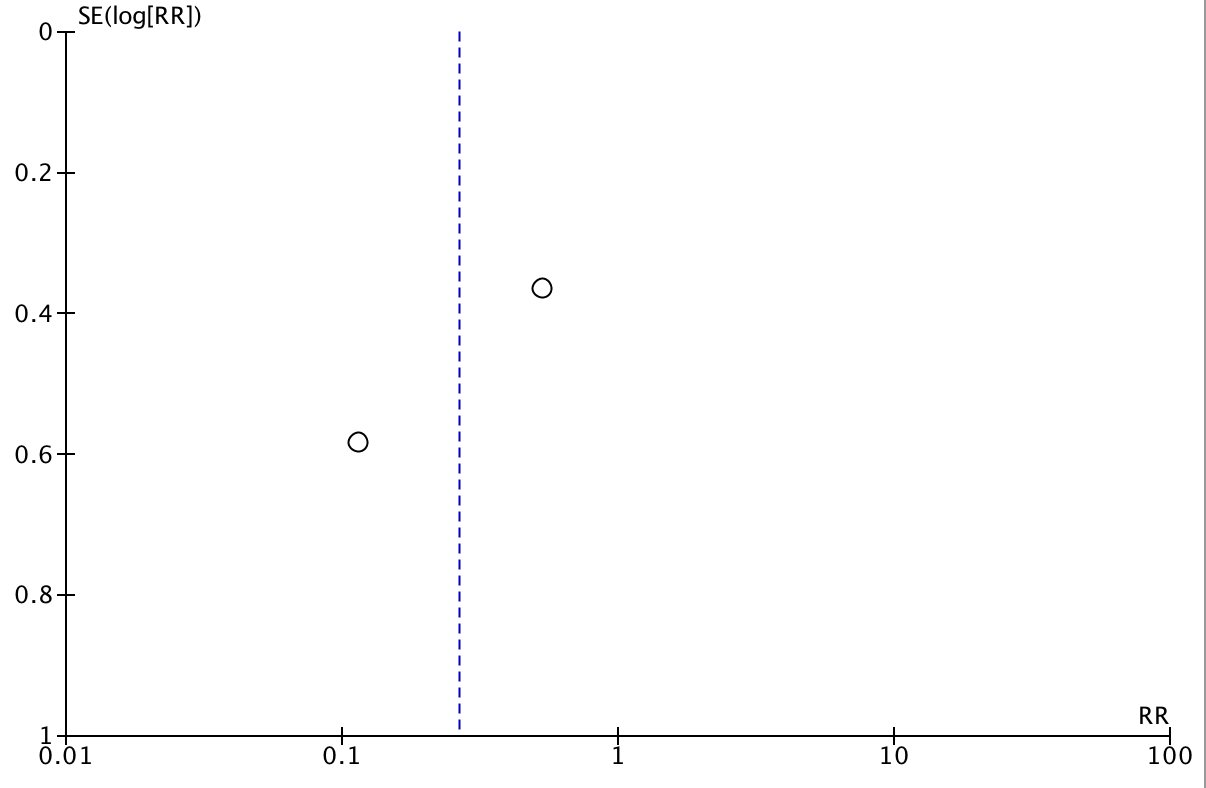

Supplement: Supplementary file 1 — Additional file 1. [file 43044_2024_558_MOESM1_ESM.docx]
